# Uncovering the Cyclic AMP Signaling Pathway of the Protozoan Parasite *Entamoeba histolytica* and Understanding Its Role in Phagocytosis

**DOI:** 10.3389/fcimb.2020.566726

**Published:** 2020-09-25

**Authors:** Shalini Agarwal, Pragyan Parimita Rath, Gaurav Anand, Samudrala Gourinath

**Affiliations:** ^1^School of Life Sciences, Jawaharlal Nehru University, New Delhi, India; ^2^International Center for Genetic Engineering and Biotechnology, New Delhi, India

**Keywords:** cyclic AMP, *Entamoeba histolytica*, phagocytosis, adenylate/adenylyl cyclase, protein kinase A, signaling

## Abstract

Second messenger signaling controls a surprisingly diverse range of processes in several eukaryotic pathogens. Molecular machinery and pathways involving these messengers thus hold tremendous opportunities for therapeutic interventions. Relative to Ca^2+^ signaling, the knowledge of a crucial second messenger cyclic AMP (cAMP) and its signaling pathway is very scant in the intestinal parasite *Entamoeba histolytica*. In the current study, mining the available genomic resources, we have for the first time identified the cAMP signal transduction pathway of *E. histolytica*. Three heptahelical proteins with variable G-protein-coupled receptor domains, heterotrimeric G-proteins (Gα, Gβ, and Gγ subunits), soluble adenylyl cyclase, cyclase-associated protein, and enzyme carbonic anhydrase were identified in its genome. We could also identify several putative candidate genes for cAMP downstream effectors such as protein kinase A, A-kinase anchoring proteins, and exchange protein directly activated by the cAMP pathway. Using specific inhibitors against key identified targets, we could observe changes in the intracellular cAMP levels as well as defect in the rate of phagocytosis of red blood cells by the parasite *E. histolytica*. We thus strongly believe that characterization of some of these unexplored crucial signaling determinants will provide a paradigm shift in understanding the pathogenicity of this organism.

## Introduction

*Entamoeba histolytica* is a major causative organism of water-borne diarrheal disease globally (Walsh, 1986; WHO, 1998). The disease accounts for nearly 50 million clinical cases and up to 100,000 deaths due to parasitic infections every year (Li and Stanley, 1996; Petri et al., 2000; Stauffer and Ravdin, 2003). *E. histolytica* displays a simple life cycle existing in two different stages, the infective cysts and vegetative trophozoites. Infection occurs when the human host ingests the infective and dormant cyst stage of the parasite through contaminated food and water. Once ingested, the cysts are converted into invasive trophozoites in the human intestine. In most infected individuals, trophozoites multiply and encyst, and the cysts thus generated pass on with the stool to infect new hosts (Haque et al., 2003; Stanley, 2003; Aguilar-Diaz et al., 2011). Majority of the amoebic infections are asymptomatic, and the parasite exists as a commensal in the gut and continues to multiply and spread. Only in a fraction of infected people (about 10%) do trophozoites invade the host tissues to cause amoebiasis. The three major steps in amoebic invasion are attachment to target tissues, cytolysis, or destruction and phagocytosis of host cells (Orozco et al., 1983; Bailey et al., 1985; Tsutsumi et al., 1992; Huston et al., 2003). Phagocytosis of the host RBCs, immune cells, dead epithelial cells, bacteria, and other unicellular organisms is an important feature of amoebic pathogenesis (Orozco et al., 1983; Tsutsumi et al., 1992). Phagocytosis of the RBCs can also lead to bloody dysentery in patients with severe intestinal invasive amoebiasis. Essentially, phagocytosis has been referred to as the key virulence marker for *Entamoeba* pathogenesis (Bracha et al., 1982). It is a complex and multistep process that requires intensive cytoskeletal remodeling and initiation of several signaling events. A number of molecules such as actin filaments, actin-binding proteins, and myosins have been identified and characterized in controlling cytoskeletal dynamics and coordinating the process of phagocytosis in *E. histolytica* (Voigt and Guillen, 1999; Voigt et al., 1999; Kumar et al., 2014a; Agarwal et al., 2019; Rath and Gourinath, 2020). The list also includes several novel proteins that have not been identified in mammalian and other systems such as transmembrane kinases and the serine-rich *E. histolytica* proteins (Boettner et al., 2008; Teixeira and Huston, 2008). Essentially, majority of the participants of the phagocytic machinery are unique to *Entamoeba* either with no homolog in other systems or with modified structures and different regulatory mechanisms. A detailed understanding of its elusive signal transduction pathway holds tremendous opportunities for understanding the evolution of this pathogen as well as for therapeutic interventions.

The genome of the parasite displays an extensive signaling network, suggesting an important role of signaling pathways in co-regulating some of these vital cellular processes (Loftus et al., 2005; Nozaki and Bhattacharya, 2014). Calcium (Ca^2+^) has evolved as one of the key second messengers in this pathogen regulating phagocytosis at multiple levels. The pathogen encodes several novel Ca^2+^ binding proteins (CaBPs), PIP_2_, IP_3_, IP_4_, and P-type Ca^2+^-ATPases, for Ca^2+^ regulation and homeostasis (Sahoo et al., 2004; Loftus et al., 2005; Jain et al., 2008; Nozaki and Bhattacharya, 2014; Sharma et al., 2019). The calcium binding protein 1 (EhCABP1) has been identified as the central indispensable molecule recruited at the early phagocytic cups. Together with EhC2PK, a C2 domain containing protein kinase and EhAK1, an atypical alpha kinase, it has been shown to modulate actin cytoskeletal dynamics during phagocytic cup formation (Somlata and Bhattacharya, 2011; Mansuri et al., 2014). The kinase EhAK1 along with other cytoskeletal proteins such as actin branching complex Arp 2/3, Myosin1B, and the calcium binding protein 3 (EhCABP3) are known to regulate the formation and progression of the phagocytic cup toward its closure (Aslam et al., 2012; Babuta et al., 2018). CaBPs (EhCaBP1, EhCaBP3, and EhCaBP5) have been shown to play a crucial role in establishment of phagocytosis in *E. histolytica* (Sahoo et al., 2004; Jain et al., 2008; Aslam et al., 2012; Kumar et al., 2014b; Nozaki and Bhattacharya, 2014; Sharma et al., 2019). However, relative to Ca^2+^ signaling, the knowledge of another crucial second messenger cAMP and its signaling pathway is very limited in *E. histolytica*. Though indirect evidence for the existence of signaling through cAMP and G-protein-coupled receptors has been documented (Soid-Raggi et al., 1998; Frederick and Eichinger, 2004; Loftus et al., 2005; Anantharaman et al., 2011; Bosch et al., 2012), the detailed signal transduction pathway has not yet been elucidated. cAMP has been shown to regulate the reorganization of actin cytoskeleton necessary for adhesion and locomotion (Ortiz et al., 2000). The high intracellular cAMP levels have been linked to up-regulation of actin mRNA transcription (Manning-Cela et al., 1997). As the parasite shows a very high rate of actin-dependent processes (phagocytosis, motility, pseudopod formation, etc.), the significance of cAMP and its signaling pathway in regulating such processes cannot be ignored. It is also quite likely that there may be crosstalk between various signaling pathways.

Henceforth, in this study we have tried to establish a proof-of-principle for the presence of functional cAMP signaling pathway in *E. histolytica*. Utilizing the available genomic resources, we have performed a comprehensive sequence as well as structural analyses to identify the major proteins and enzymes of the cAMP signaling repertoire. Three genes encoding GPCR-related domains were identified, which are unique to this parasite. Also prominent within its genome are all the three G-protein subunits (Gα, Gβ, and Gγ subunits) that, when activated by GPCR, are known to transduce the signals for cAMP formation. Looking for interacting partners of Gα protein, we could not identify any transmembrane adenylyl cyclase (tmAC) in its genome. However, we mined a single distinctive soluble adenylyl cyclase (sAC) in its genome and one cyclase-associated protein (CAP) containing the conserved cyclase-binding motif. A single gene encoding carbonic anhydrase (CA), phylogenetically similar to other lower eukaryotes, was also evident in its genome. Our search also resulted in several putative genes for the two key cAMP effectors: protein kinase A (PKA) and exchange protein directly activated by cAMP (EPAC). A diverse set of cytoplasmic scaffolds often found associated with cAMP signaling cascade such as A-kinase anchoring proteins (AKAPs) and Rap GTPases for the respective regulation of PKA and EPAC were also evident. We next examined the effect of known modulators of some of these key components in *Entamoeba* trophozoites (Manning-Cela and Meza, 1997; Manning-Cela et al., 1997; Paveto et al., 1999; Dawn et al., 2014). The modulation of heterotrimeric G-protein and AC signaling pathway by the pharmacological agent forskolin (FK) resulted in increased intracellular levels of cAMP and enhanced phagocytic activity in trophozoites, whereas the widely used manipulators of the cAMP responsive protein kinase (H-89) and EPAC (ESI-09) pathway reduced phagocytosis significantly. The amenability of pathway components to pharmacological manipulation further endorsed our hypothesis for a functional cAMP signaling system in *E. histolytica*. Thus, overall, our study provides valuable insights into the cAMP signaling pathway in *E. histolytica* that warrants further experimental investigations to better understand the functional roles of individual components.

## Materials and Methods

### Parasite Culture and Maintenance

*E. histolytica* trophozoites (HM1:IMSS strain) were grown and maintained axenically in TYI-S-33 medium supplemented with 15% adult bovine serum, 1 × Diamond's vitamin mix, and antibiotic (125 μl of 250 U/ml benzyl penicillin and 0.25 mg/ml streptomycin per 100 ml of medium) as described by Diamond et al. (1978).

### Data Mining and Bioinformatics Analysis

We performed the *E. histolytica* genome and proteome analysis using both sequence and structural homology approaches. The cAMP signaling begins with a transmembrane GPCR followed by G-proteins, adenylate cyclase (tmAC, sAC), adenylyl CAP, CA, PKA, EPAC, phosphodiesterase (PDE), AKAP, and Rap GTPases. At first, we scanned the Interpro (Mitchell et al., 2019) database and retrieved the respective domains corresponding to each of the abovementioned proteins of the pathway. The advantage of using Interpro is that it takes into account a single member protein class as well. This database has all the algorithms combined from various sequence analysers like Pfam, PRINTS, SUPERFAMILY, etc. With the above identified domain sequences, we then searched the *E. histolytica* (Taxonomic ID: 5759) proteome downloaded from the UniProt database (UniProt, 2019). The resulting sequences from the domain search were then tabulated and analyzed to remove the redundant and low complexity sequences. To refine our search further, we next performed a structural similarity search using the Phyre2 server (Kelley et al., 2015). In this approach, we submitted each selected sequence for homology modeling, matching the closest homologous structures deposited in the PDB (Protein Data Bank). With this kind of strategy, we were able to perform a two-step identification of the protein that takes into account both the sequence and structure-based searches. Except for PDE, we found several hits for the presence of relevant domains in a number of amoebic proteins. Due to the low sequence identity issue with PDE, we reversed our search protocol. First, we tabulated the PDE present in humans and used those sequences in the Delta-BLAST program of the BLASTp suite (Altschul et al., 1990). With this approach, we were able find few probable PDE in *E. histolytica*. All the proteins that we were able to characterize with these *in silico* studies are provided in Table 1.

**Table 1:** List of all the elements of the cAMP signaling pathway identified from *E. histolytica*.

**Name of the protein**	**UniProt ID**	**AmoebaDB ID**
**G-protein-coupled receptor**		
ABA GPCR	C4LVA8/A0A175JGJ9	EHI_196900
Rhodopsin-like GPCR	C4LSB5/A0A5K1V1E8	EHI_153230
GPCR like receptor	C4M112/A0A5K1U9Q6	EHI_105070
GPCR GABA-B	C4LWI0/A0A175JHL1	EHI_096680
**Adenylate cyclase**		
Adenylate Cyclase	C4M560/A0A5K1UEB1	EHI_178070
**Adenylate cyclase-associated protein (CAP)**		
CAP C-terminal	B1N3T9/A0A175JT55	EHI_081430
CAP	C4M295/A0A5K1VRV0	EHI_136150
**Carbonic anhydrase**		
Carbonic anhydrase	C4LXK3/A0A5K1UAB3	EHI_073380
**A-kinase associated protein**		
Rab family GTPase	Q5NT19/A0A175JPA5	EHI_169090
Rab family GTPase	Q5NT05/A0A5K1VDR2	EHI_177520
**Protein kinase A**		
Protein kinase A1	Q8WQH7/A0A5K1UPF8	EHI_004790
Protein kinase A2	C4LX83/A0A5K1V725	EHI_013170
Protein kinase A3	C4M4G9/A0A5K1TZC2	EHI_042150
Protein kinase A4	Q26334/A0A5K1UQZ7	EHI_053130
Protein kinase A5	C4M7B3/A0A5K1VQ71	EHI_055710
Protein kinase A6	C4M546/A0A5K1UL68	EHI_078200
Protein kinase A7	C4MBR0/A0A5K1VTH1	EHI_131660
Protein kinase A8	C4M2W0/A0A5K1VFW0	EHI_188930
**G Proteins**		
G-protein alpha	A0A5K1VRJ6	EHI_140350
G-protein beta	A0A5K1UYB7	EHI_000240
G-protein gamma	A0A175JMJ3	CL6EHI_c00092
G-protein beta-like1	A0A5K1V7W5	CL6EHI_171280
G-protein beta-like2	A0A5K1VM61	CL6EHI_110400
G-protein beta-like3	A0A5K1VNH1	CL6EHI_050550
**Exchange protein activated by cAMP**		
EPAC	A0A175JLB5	EHI_134820
EPAC	A0A5K1VCL2	EHI_180310
EPAC	A0A5K1U0E5	EHI_069530
EPAC	A0A5K1V3V3	EHI_152140
EPAC	A0A5K1TWX1	EHI_072110
EPAC	A0A5K1VSI0	EHI_197190
EPAC	A0A5K1V479	EHI_110240
EPAC	A0A5K1V0B4	EHI_135780
EPAC	A0A5K1TVB9	EHI_086010
EPAC	A0A5K1V653	EHI_138430
EPAC	A0A5K1UFF4	EHI_181600
EPAC	A0A5K1VJC5	EHI_169080
EPAC	A0A5K1UDN2	EHI_100380
EPAC	A0A5K1UI03	EHI_013140
EPAC	A0A5K1V1G3	EHI_148500
EPAC	A0A5K1U3C1	EHI_067890
EPAC	A0A5K1USG1	EHI_016400
EPAC	A0A5K1U012	EHI_152200
EPAC	A0A5K1UX04	EHI_100390
EPAC	A0A175JGX7	EHI_142220
EPAC	A0A5K1V1X2	EHI_183450
EPAC	A0A175JNI7	EHI_030740
EPAC	A0A175JTP8	EHI_070760
EPAC	A0A5K1UBN8	EHI_167170
EPAC	A0A5K1VUR6	EHI_170600
EPAC	A0A5K1UC90	EHI_023270
EPAC	A0A5K1ULD4	EHI_158330
EPAC	A0A5K1V3Z2	EHI_092420
EPAC	A0A5K1UBG4	EHI_068110
EPAC	A0A5K1UB02	EHI_113430
**Phosphodiesterase**		
PDE-like	C4LUA0/A0A5K1UBD8	EHI_110510A
PDE-like	B1N2Y8/A0A5K1UL04	EHI_035380A
PDE-like	C4LZN2/A0A5K1UYI4	EHI_175070A
PDE-like	C4M3K4/A0A5K1VCI3	EHI_109850A
PDE-like	C4MAL3/A0A5K1U7X4	EHI_123230A
PDE-like	C4M411/A0A5K1UVP9	EHI_195060A
**Rap GTPase**		
Rap1/EhRap1	A0A175JZ29	EHI_049030
Rap1a	A0A175JSN7	EHI_119300
Rap	A0A5K1VC31	EHI_198330
Rap	A0A5K1TVC1	EHI_154240
Rap	A0A5K1VF21	EHI_124520
Rap1a	A0A5K1UU74	EHI_051090
Rap1a	A0A5K1UDF7	EHI_015350
Rap/EhRap2	A0A5K1VPQ0	EHI_058090
Rap1a	A0A5K1UDK1	EHI_068120
Rap2b	A0A5K1UWT4	EHI_109840
Rap2a	A0A5K1UBR2	EHI_178900
Rap	A0A5K1VAR0	EHI_170140

*The table includes the name of the protein and their corresponding UniProt and AmoebaDB ID*.

In order to trace the evolutionary history of amoebic proteins, we tried to find homologous proteins in other organisms. We used the simple BLASTp for all our sequences. However, this strategy failed to identify any proteins from the known model organisms. Moreover, the majority of proteins that we found were hypothetical or putative in nature, barring a few. Additionally, the identity percentage was extremely less. Thus, to improvise our search, we also used Delta-BLAST, but we did not find any major identical protein to these amoebic proteins. Our search had already included the structural similarities when we used the homology modeling approach through the Phyre2 server. Finally, we used the homologous sequences from different organisms that have been characterized earlier and tried to study evolution through these. We aligned all the sequences using the ClustalW program. To analyze the sequence alignments and phylogenetic relations, we used MEGA 7 software (Kumar et al., 2016). The final tree images were prepared with iTOL (Letunic and Bork, 2019).

### Measurement of Cytoplasmic cAMP Levels

Cytosolic levels of cAMP were measured in *Entamoeba* trophozoites by using the cAMP direct immunoassay kit (Calbiochem, USA) as per the manufacturer's protocol. We used FK, a widely used cAMP-elevating agent at a concentration of 100 μM, at which it has earlier been shown to consistently induce cAMP levels in *Entamoeba* and other parasites (Paveto et al., 1999; Frederick and Eichinger, 2004; Swierczewski and Davies, 2009). Approximately 2 × 10^5^ trophozoites were harvested per tube, rinsed with PBS, pH 7.2, and incubated with 100 μM FK (Sigma) or DMSO as control for 30 min at 37°C in six-well-plates in triplicate. A standard curve was generated using cAMP standards provided in the kit. Following incubation, cells were harvested and lysed, and quantitative measurement of cAMP was obtained from the equation derived from the standard curve. Total protein content of the trophozoite lysate was determined by BCA (Pierce BCA Protein Assay Kit), and the amount of cAMP/mg of protein was determined in each sample. One-way analysis of variance was performed for determining statistical significance (*P* < 0.005). All calculations of statistical significance were made using the Sigma plot software and GraphPad prism.

### Erythrophagocytosis Assay

For monitoring erythrophagocytosis, *E. histolytica* trophozoites were harvested in serum-free TYI-33 medium and RBCs were obtained in PBS, pH 7.4, via needle prick of the middle finger (Agarwal et al., 2019). The PKA inhibitor (H-89), recently identified EPAC inhibitor (ESI-09), and FK were each used at the optimal concentrations at which they have been used in previous studies in other parasites (Ydrenius et al., 2000; Kurokawa et al., 2011; Almahariq et al., 2013; Dawn et al., 2014; Jia et al., 2017). Approximately 5 × 10^5^ trophozoites were harvested per tube, rinsed with PBS, pH 7.2, and incubated with 100 μM FK (Sigma), 100 μM H-89 (Sigma), 100 μM ESI-09 (Sigma), or DMSO as control for 30 min at 37°C. Following treatment, cells were washed and incubated with RBCs for varying time points ranging from 10 to 40 min at 37°C in 1 ml of incomplete TYI-33 medium. Cells were then collected and centrifuged, and non-engulfed RBCs were lysed by the addition of chilled distilled water and removed by centrifugation at 1,000 g for 5 min. Amoebic cells were then resuspended in formic acid to lyse trophozoites containing engulfed RBCs. The absorbance for RBCs taken up by trophozoites was then measured at 400 nm using formic acid as Blank by spectrophotometer (Multiscan Go, Thermo Scientific). Statistical comparisons were made using two-way ANOVA test (*P* < 0.0001). All calculations of statistical significance were made using the Sigma plot software and GraphPad prism.

### TRITC-Phalloidin Staining and Immunofluorescence Assay

Briefly, *E. histolytica* trophozoites following treatment with 100 μM FK, 100 μM H-89, 100 μM ESI-09, or DMSO as control for 30 min at 37°C were harvested by low-speed centrifugation and incubated with RBCs for 10 min. Cells were then collected and transferred to 8-mm round well dishes on a slide glass, fixed with 3.7% pre-warmed paraformaldehyde (30 min), and permeabilized with 0.2% Triton X-100/PBS (3 min). Fixed cells were then quenched in 50 mM NH_4_Cl (30 min) and then blocked in 1% BSA/PBS (2 h). BSA-incubated cells were then washed and probed with TRITC-Phalloidin at 1:250 for 1 h). The preparations were further washed with PBS and mounted on a glass slide using DABCO [1,4-diazbicyclo (2,2,2) octane (55) 2.5% in 80% glycerol]. Images were acquired in confocal microscope (A1R, Nikon, Japan).

## Results

### Identification of Enzymes and Effectors of cAMP Signaling Pathway in *E. histolytica*

**Figure 1:**
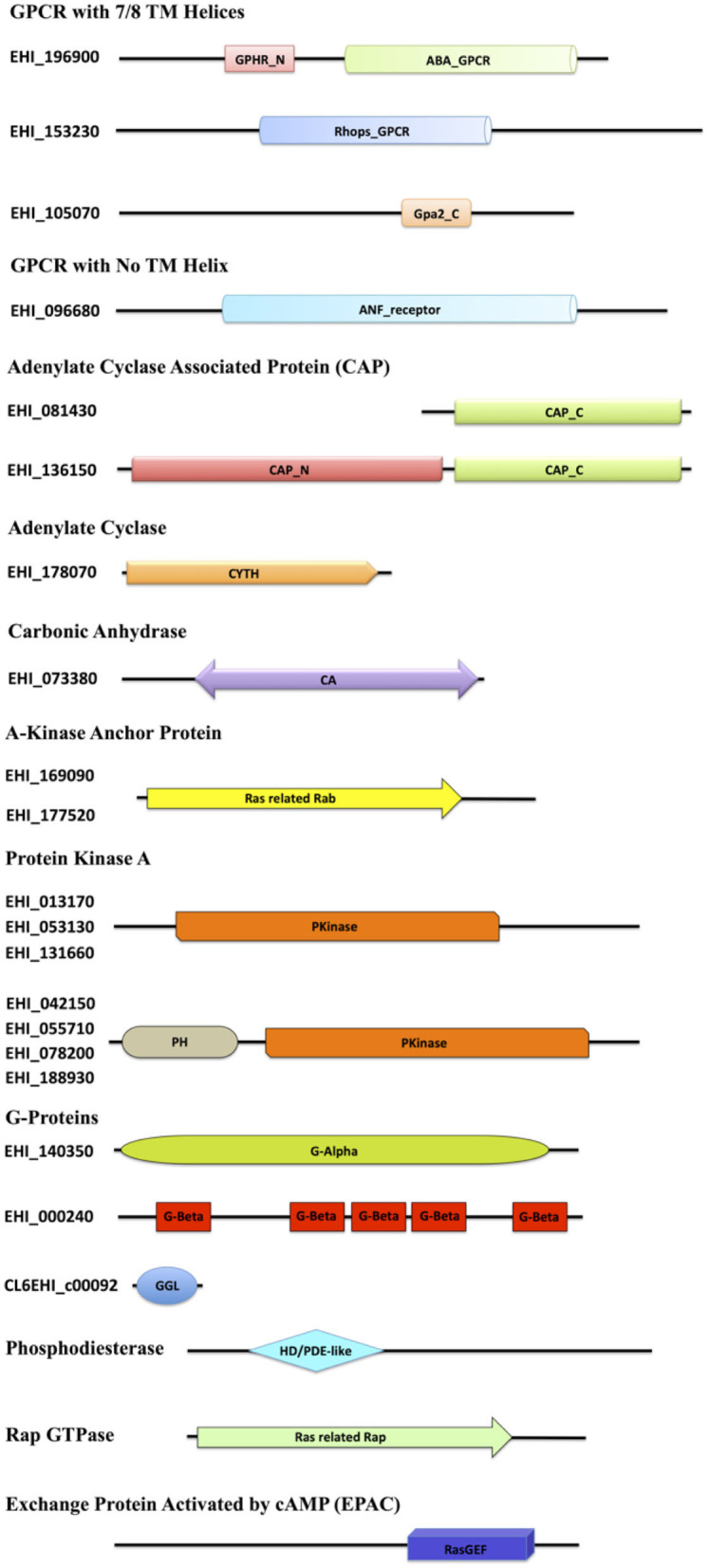
Diagrammatic representation of the domain architecture of the cAMP signaling proteins from *E. histolytica*. The domain abbreviations used in the diagram are as follows: ABA_GPCR, abscisic acid G-protein-coupled receptor; GPHR_N, Golgi pH Regulator (GPHR) Family N-terminal; Rhops_GPCR, rhodopsin-like GPCR transmembrane domain; Gpa2_C, G-protein-coupled glucose receptor regulating Gpa2 C-terminal; ANF_receptor, receptor family ligand binding region; TM, transmembrane; CAP_C, C-terminal domain of adenylyl cyclase-associated protein; CAP_N, N-terminal domain of adenylyl cyclase-associated protein; CYTH, domain present in the members of the adenylate cyclase family; CA, carbonic anhydrase; Ras related Rab, protein similar to Rab29/Rab38/Rab32, commonly serving as an AKAP; PH, pleckstrin homology domain; Pkinase, protein kinase domain; G-Alpha, G-protein alpha; G-Beta, G-beta repeats (WD40); GGL, G-protein gamma-like; HD/PDE-like, phosphodiesterase-like; Ras-related Rap, Rap GTPase; RasGEF, Ras Guanine exchange factor domain.

The *E. histolytica* genome has been completely sequenced, providing many insights into its complex biology (Loftus et al., 2005). Although many features of the eukaryotic signaling system are retained in its genome, a vast array of components has no homologs in other systems. Mining the available genomic and proteomic resources using two distinct search strategies, we have in this study enlisted various elements of the cAMP signaling pathway of this pathogen (Table 1). The corresponding domain structures for each of the identified protein, based on analysis of the UniProt primary sequences, have been represented in Figure 1. cAMP signaling in general is known to involve a series of steps starting from the sensing of an external stimuli to the activation of cell surface receptor proteins such as GPCRs. GPCRs couple to heterotrimeric G-proteins and modulate the activity of ACs to signal through secondary messengers like cAMP.

As of now, no GPCRs have been discovered in *Entamoeba*, except for a putative GPCR (EhGPCR-1) described by Picazarri et al. (2005), Bosch et al. (2012). However, sequence analysis and NCBI domain database search for EhGPCR-1 revealed that it is more closely related to a Wnt-binding factor and lacks any GPCR signature domains (Bosch et al., 2012; Bosch and Siderovski, 2013). We therefore first scanned the *Entamoeba* genome for identifying putative GPCR-like genes. We used the domain-based search strategy and found three heptahelical proteins resembling the traditional GPCRs, A0A175JGJ9, A0A5K1V1E8, and A0A5K1U9Q6, with variable GPCR domains. A0A175JGJ9 consisted of two domains, GPHR_N and ABA_GPCR, both of which are commonly found in the G-type abscisic acid GPCRs and Golgi pH regulators (Maeda et al., 2008; Pandey et al., 2009). A0A5K1V1E8 harbored a domain belonging to the Rhodopsin superfamily of GPCRs (Figure 1) (Tsukada et al., 2003). The third protein, A0A5K1U9Q6, with its Gpa2_C domain, was found to be a member of the glucose-sensing GPCR protein GPR1 (Xue et al., 1998). Similarly, in one of the protein A0A175JHL1, we did not find any TM helix; it did contain an ANF receptor ligand-binding domain (known to bind several extracellular ligands) (Kuryatov et al., 1994). In conjunction with this search, our structural analysis yielded that the protein matches the GABA-responsive GPCR, which commonly contains the ANF domain (Kumar et al., 2016). However, the GPCR sequences identified within these families shared <25% identity with other related species.

The signal from GPCRs is known to pass onto G-proteins, and studies have earlier identified functional heterotrimeric G-protein subunits (Gα, Gβ, and Gγ) in *E. histolytica* required for key pathogenic processes (Loftus et al., 2005; Bosch et al., 2012; Nozaki and Bhattacharya, 2014). We also found one of each type, Gα (A0A5K1VRJ6), Gβ (A0A5K1UYB7), and Gγ (A0A175JMJ3), along with other three proteins that may act as distant relatives of Gβ (A0A5K1V7W5, A0A5K1VM61, and A0A5K1VNH1). We could not identify any tmAC-encoding gene downstream to G-protein signaling. However, since a transmembrane or a cytoplasmic soluble AC produces cAMP in eukaryotic cells, we looked for sAC encoding gene as well. A single, distinctive sAC (A0A5K1UEB1) was mined from the amoeba proteome with the canonical CYTH domain. This domain is predominantly found in a novel superfamily of bacterial ACs and mammalian thiamine triphosphatases, serving as a link between prokaryotic and eukaryotic homologs (Iyer and Aravind, 2002). Further, we looked for the next member of the pathway, CAP. We found one full-length protein (A0A5K1VRV0) and one C-terminal fragment (A0A175JT55). The amoebic CAP had the conserved RLEXAXXRLE motif A at the N-terminal, known to interact with ACs (Hubberstey and Mottillo, 2002). Functionally, the N-terminal domain in CAPs is recruited in cellular signal relay through cAMP, while the C-terminal domain is required to modulate actin dynamics (Hubberstey and Mottillo, 2002). The conservation of adenylate binding site in EhCAP, despite the lack of tmAC in its genome, does point out toward an alternate AC signaling route in this parasite. However, the specificity of this motif for the only identified sAC in its genome or not-yet-identified tmAC warrants further investigation. sACs have only been a recently recognized source of cAMP signaling, and unlike tmACs, they are in general regulated by bicarbonate ions in the cells that are fine-tuned by the presence of the enzyme CA. CA catalyzes the reversible hydration of carbon dioxide and water into carbonic acid, protons, and bicarbonate ions. The bicarbonate ions act as the activators of sAC and thus, bridges the enzyme CA to this signaling pathway. We, therefore, further looked for the presence of enzyme CA and found a single gene (A0A5K1UAB3) for this enzyme with the CA domain, belonging to the β-class family.

Downstream to cAMP production, several putative candidates for the two key cAMP-activated intermediaries between ACs and the regulatory events, PKA and EPAC, were mined from its genome. *E. histolytica* encodes 24 proteins in the AGC kinase family (Anamika et al., 2008). However, with our structural homology search, we could classify eight of them as PKA. Further experimental validation will help to completely characterize them. Our search also concluded 12 Rap GTPases, which contain the Ras related Rap domain found in all homologs. We found an abundance of proteins in our search that contained the RasGEF domain, but with our two distinct search approach of using sequence as well as structural similarity, we could identify 30 EPAC among them (Table 1, Figure 1). The conventional PDEs seem to be missing in the amoeba proteome; however, we managed to find six proteins with the PDE-like domain (Table 1, Figure 1).

### Phylogenetic Evolution of the Pathway Components in *E. histolytica* and Other Protist Parasites

To explore the evolutionary history of the identified key effectors of the cAMP pathway in *E. histolytica*, we first tried to search their homologs in human, yeast, and other protist parasites. *E. histolytica* is known for the presence of unique proteins in its proteome, and thus, many a times they do not yield any homologs during the BLASTp searches. In our study as well, the blast searches for GPCRs, AC, and PDE yielded no significant homologs in other systems. The sequences retrieved for *Entamoeba* were very different and unique, and we could not find their related proteins in either of the organisms included in this study (*Homo sapiens, Drosophila melanogaster, Dictyostelium discoideum, Saccharomyces cerevisiae, Giardia lamblia, Trypanosoma cruzi, Plasmodium falciparum*, and *Leishmania donovani*). Few similar proteins were pulled out during the BLASTp search with the GPCR sequences in other distantly related organisms, but with no significant identity (below 23%) that could be used to trace the evolutionary course. For example, in case of A0A175JGJ9 (ABA_GPCR domain), the maximum hits were type G GPCRs from plants, although their identity was still below 23%. Similarly, when we subjected the adenylyl cyclase sequence for homology search, we only retrieved bacterial CYTH domain proteins. We found none of the eukaryotic homologs, which makes us believe that the adenylyl cyclase is an acquired gene from the prokaryotic origins, projecting the evolutionary lineage of *Entamoeba* between prokaryotes and eukaryotes. Searching PDE in amoeba was the most challenging of all, since none of the known PDE proteins matched with the proteins of this pathogen. The few PDE-like molecules, the best we could find in *Entamoeba*, had no significant search hits with other proteins from the organisms listed above.

**Figure 2:**
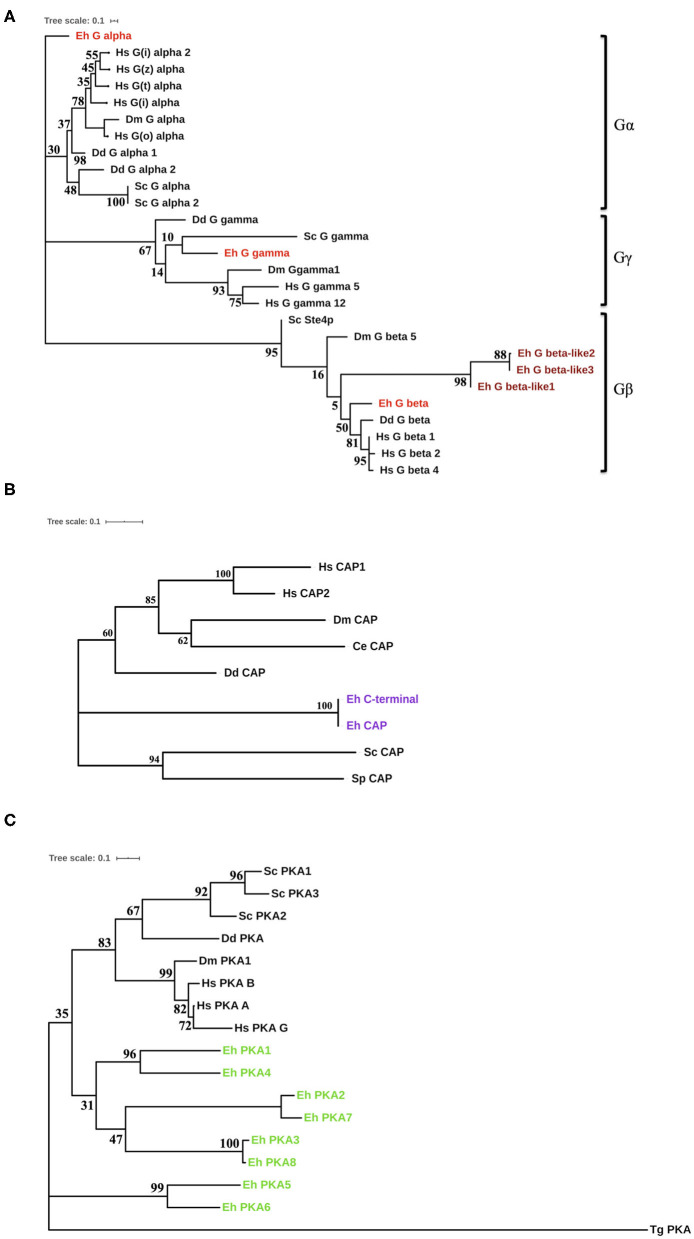
Phylogenetic analysis of the cAMP pathway components from *E. histolytica*. **(A)** Phylogenetic analysis of the G-proteins. Three G-proteins were identified in *E. histolytica* (Eh): Gα (G alpha), Gβ (G beta), and Gγ (G gamma). The sequences of these putative proteins were analyzed phylogenetically with G-proteins from other organisms. Hs, *Homo sapiens*; Dm, *Drosophila melanogaster*; Dd, *Dictyostelium discoideum*; Sc, *Saccharomyces cerevisiae*. The amoebic homologs are highlighted in red color (maroon for the diverged amoebic proteins). **(B)** Phylogenetic tree for cyclase-associated proteins. The cyclase-associated proteins from *E. histolytica* branches out as a separate node in the phylogenetic tree marked purple. However, it contains all the conserved motifs present in CAPs across phyla. **(C)** Evolution of protein kinase A. The eight identified PKA from *E. histolytica* fall on a separate branch in the evolutionary tree. This tree has a maximum log-likelihood of −2261.18. *Toxoplasma gondii* (Tg), added to the list of earlier analyzed organisms, had the most diverged PKA, while others shared one node.

For the proteins (G-proteins, CAP, CA, and PKA) for which we found homologs in non-*Entamoeba* systems, we constructed an un-rooted phylogenetic tree using the maximum-likelihood method (Figures 2A–C, Supplementary Figure 1). The initial trees were generated by the neighbor-joining method and BioNJ algorithm, which were then subjected to an extensive heuristic search. The pairwise distance matrix applied to these sequences was based on the Jones–Taylor–Thornton model. Finally, the topology with maximum log value was selected for further analysis. In case of the G-proteins, we were able to construct a phylogenetic tree with the highest log-likelihood of −2362.91 (Figure 2A). The tree clearly formed three branches, each corresponding to Gα, Gβ, and Gγ. Gα diverged out very early in evolution, while Gβ had higher similarity with the human homologs. Gγ, on the other hand, exhibited close relation with the yeast protein. The evolutionary history places the CAP sequences from *E. histolytica* close to the *Dictyostelium discoideum* counterpart with a maximum of 39% identity (Figure 2B). However, they still separated out as a different node on the tree (log-likelihood −2261.18). The sole CA coded by *Entamoeba* is phylogenetically similar to other lower eukaryotes (Supplementary Figure 1). From the nine sequences analyzed, a total of 148 positions were traced in the final dataset. The phylogenetic tree for amoebic PKA was one of a kind (Figure 2C). The log-likelihood of the un-rooted tree was −7178.08. The PKA sequences from *Entamoeba* bifurcated from the node separating them from the other sequences. These data were analyzed from a total of 289 positions in the analyses. While PKA from *Toxoplasma gondii* formed the most distant branch, the amoebic proteins branched out in pairs on the tree. PKA5 and PKA6 were located on a different branch altogether; thus, they were the most distant ones among all PKAs from *E. histolytica*. All these search results strongly indicate that several of the components of cAMP signaling pathway are unique to this pathogen. Either they have no homologs in other systems or they have modified structures and thus their specific characterization warrants further in-depth experimental investigations.

### Pharmacological Manipulations of cAMP Pathway Components and Their Role in Phagocytosis

**Figure 3:**
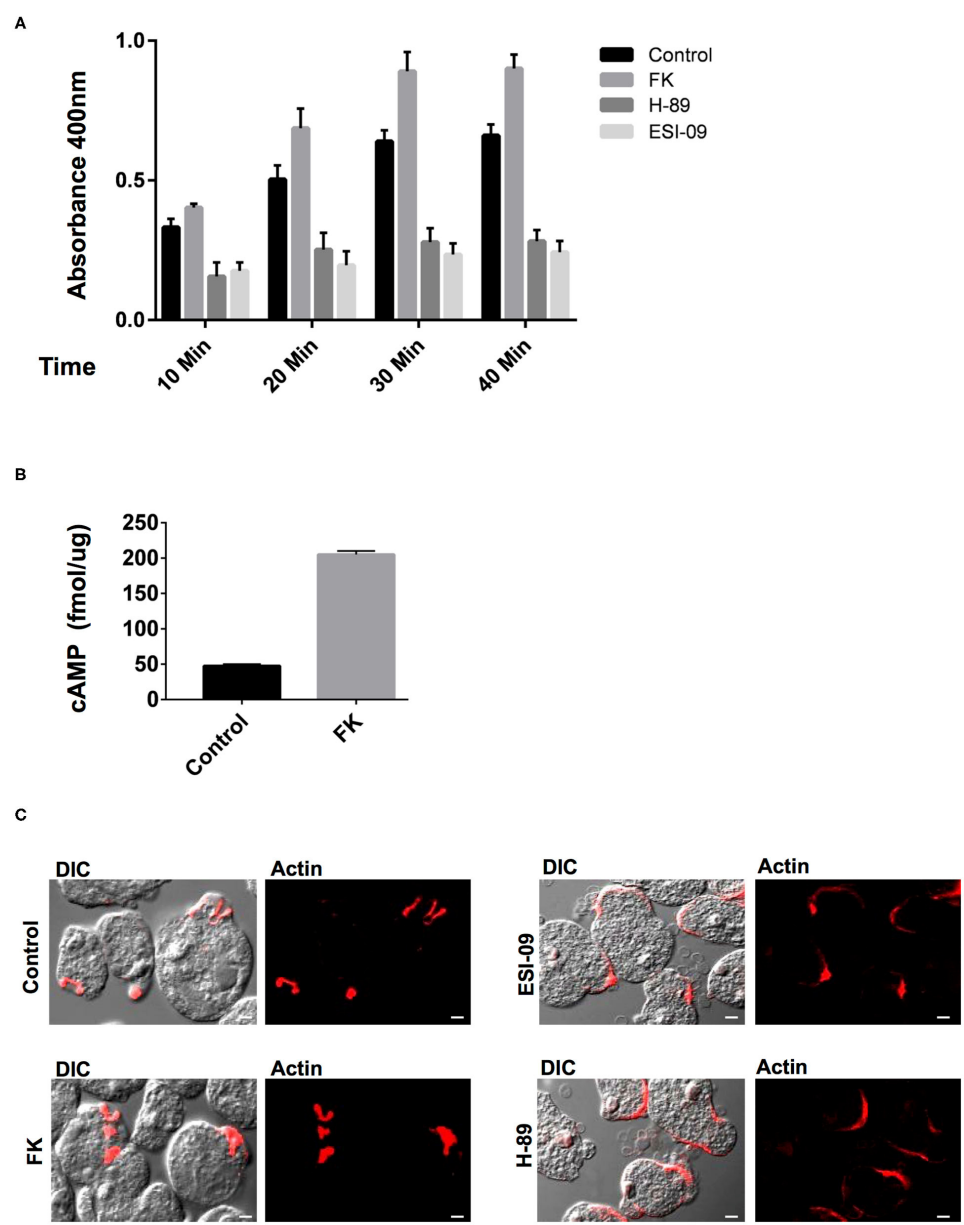
Pharmacological manipulation of the elements of cAMP signaling pathway and their role in phagocytosis. **(A)** The graph represents the spectrophotometer reading for RBC uptake by *E. histolytica* cells pre-treated with either Forskolin (FK), H-89 and ESI-09, or DMSO control at 10, 20, 30, and 40 min. The experiments were repeated independently three times in duplicate with error bars indicating the standard error. Statistical comparisons were made using two-way ANOVA test (*P* < 0.0001). **(B)** The graph represents the cAMP levels in Forskolin (FK) and DMSO control cells as measured via a spectrophotometer. The experiments were repeated independently three times in duplicate with error bars indicating the standard error (*P* < 0.005). **(C)** Visualization of RBC phagocytosis in treated and control cells. Cells pre-treated with specific modulators as mentioned above were incubated with RBCs and fixed and stained for actin with TRITC-Phalloidin. Image panels indicate TRITC-Phalloidin-stained cells labeled as Actin and DIC for differential interference contrast (scale bar, 10 μm).

Earlier studies on the effects of typical GPCR and G-protein agonists have documented perturbation of virulence and pathogenicity in *Entamoeba* (Khan and Sen, 1988; Khan et al., 1990; Bosch et al., 2012). Moreover, the high intracellular cAMP levels have been linked to actin cytoskeleton rearrangements, up-regulation of its actin mRNA transcription, and stage transition events (Manning-Cela et al., 1997; Ortiz et al., 2000). These evidences for the pharmacological sensitivity of G-protein signaling encouraged us to further explore the specific modulators of the downstream cAMP pathway (Brauner-Osborne et al., 2007). Pharmacological agents such as forskolin (FK) are a widely used activator of adenylate cyclase and thereby inducer of intracellular cAMP levels (Manning-Cela and Meza, 1997; Paveto et al., 1999; Kirkman et al., 2001; Swierczewski and Davies, 2009). In *Entamoeba* cells as well, earlier studies with FK have resulted in stimulation of cAMP levels and modulation of actin cytoskeleton dynamics (Manning-Cela and Meza, 1997; Paveto et al., 1999; Frederick and Eichinger, 2004). FK has also been shown to enhance adenylate cyclase activity and stimulate phagocytosis in *Paramecium* (Wyroba, 1987). We therefore, validated the effect of FK on cAMP manipulation at the same concentration of 100 μM, at which it consistently manipulates cAMP levels in *Entamoeba* and other parasites (Paveto et al., 1999; Frederick and Eichinger, 2004; Swierczewski and Davies, 2009). Addition of FK to the trophozoites resulted in increase in cAMP levels that, when analyzed as compared to DMSO-treated cells, was almost 4-fold enhanced (Figure 3C). The FK-treated trophozites were also examined for their ability to phagocytose RBCs via erythrophagocytosis assay. The erythrophagocytosis assay were first standardized and quantified in untreated trophozoites incubated with RBCs at different time points (Supplementary Figure 2). FK-treated cells along with DMSO control cells were then evaluated in a similar condition at the same time points for their phagocytic uptake of RBCs (Figure 3A). Enhanced RBC uptake was observed in these cells with increasing incubation time (10–40 min) as compared to control cells (Figure 3A). Nicely formed phagocytic cups with accumulation of F-actin were also clearly visible in these cells (Figure 3B) similar to control cells when stained with TRITC-phalloidin and imaged on confocal microscope. However, the increase in rate of phagocytosis as compared to the significantly enhanced cAMP levels observed in trophozoites upon FK treatment was quite less. It is likely that FK-induced cAMP levels in the cells might activate multiple downstream signaling pathways. The spatiotemporal characteristics of artificially induced cAMP levels and how this accompanies the process of phagocytosis need further understanding. Nevertheless, the responsiveness of trophozites to FK in the form of up-regulated cAMP levels and enhanced phagocytic activity does endorse for a cAMP-dependent signaling mechanism in the control of phagocytosis (Figure 3B).

In general, the signals from the cAMP-activated AC are relayed onto the downstream effectors and finally to the regulatory events in the cell. The PKA and EPAC are the two key cAMP-activated intermediaries between ACs and downstream regulatory events in cAMP signaling pathway (Ydrenius et al., 2000; Kurokawa et al., 2011; Almahariq et al., 2013; Dawn et al., 2014; Jia et al., 2017). Inhibition of PKA activity by H-89 has also been linked to reduced phagocytosis in human neutrophils and decreased differentiation and replication in *Toxoplasma* parasites (Ydrenius et al., 2000; Kurokawa et al., 2011). We therefore next examined the effects of specific inhibitors of these key downstream effector proteins for cAMP: PKA (H-89) and EPAC (ESI-09) on *Entamoeba* trophozoites phagocytic activity. We found that treatment of trophozoites with either H-89 or ESI-09 caused a marked reduction in RBC uptake by these cells as compared to the DMSO-treated control cells (Figure 3A). Moreover, when these cells were visualized for phagocytic cup formation and F-actin staining, majority of the RBCs were found attached at the surface in cluster with very few cells forming phagocytic cups. Partial staining of F-actin at the RBC attachment site was observed in these cells when they were visualized for TRITC-phalloidin (Figure 3C). The *in vitro* observations thus suggest an important role of cAMP signaling in phagocytosis as well as in the dynamics of F-actin rearrangement during the process.

## Discussion

**Figure 4:**
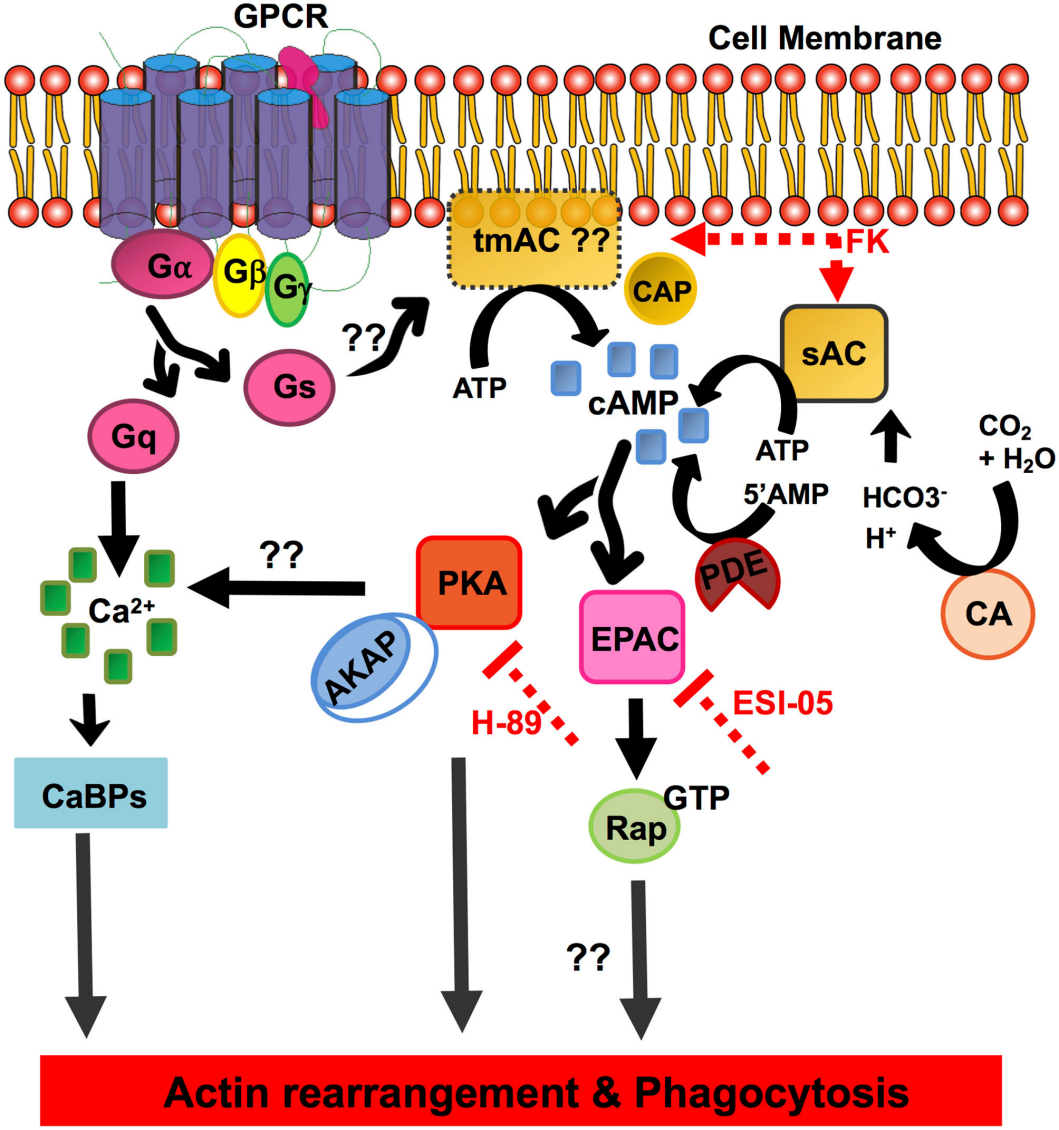
Schematic overview of the elements of the cAMP signal transduction pathway identified in *E. histolytica*. The diagram depicts the pathway components involving cAMP activation, formation/degradation, and downstream effector proteins important for cAMP signaling. GPCR, G-protein-coupled receptors; heterotrimeric G-proteins, Gα, Gβ, and Gγ subunits; Gs, stimulatory G-protein; Gq, G-protein subunit that activates phospholipase C; tmAC, transmembrane adenylate cyclase; sAC, soluble adenylate cyclase; cAMP, cyclic AMP; CAP, adenylate cyclase-associated protein; CA, carbonic anhydrase; FK, forskolin; PDE, phosphodiesterase; PKA, protein kinase A; EPAC, exchange protein activated by cAMP; AKAP, A-kinase anchoring proteins; Rap-GTP, GTP-binding Ras-related protein; H-89, inhibitor of PKA; ESI-09, inhibitor of EPAC; CaBPs, calcium-binding proteins.

*E. histolytica* displays diverse and unique signaling networks when compared to its closely as well as distantly related species. Although many features of the eukaryotic signaling system are retained in its genome, vast arrays of components are uniquely present only in this organism with no homologs in other systems. It is for one of these reasons many of its phagocytic receptors and signaling pathways remain unknown. Since the cAMP signaling system has not yet been determined in *E. histolytica*, through a thorough *in silico* analysis we have in this study unraveled its cAMP signal transduction pathway (Figure 4).

Our *in silico* analysis predicted the presence of three heptahelical proteins with a variable GPCR domain, none of which have been functionally characterized to date. The identified GPCR sequences and the receptor ligand-binding domains shared <25% identity with other related species. Downstream to GPCRs, the core heterotrimeric Gα, β, and γ subunits were found conserved in its genome with significant homologs in other systems. A number of putative candidates for major cAMP-effector proteins such as PKA and EPAC were also mined from its genome. Other proteins often found in cAMP signaling cascades, such as AKAP, Rap, and Ras, were also identified in this parasite. While evidences for the transmembrane AC sequences could not be retrieved, our *in vitro* studies with the addition of AC-stimulating compound forskolin promoted cAMP accumulation and enhanced phagocytic activity in *Entamoeba* trophozoites (Figure 3). Nevertheless, we identified a single, distinctive soluble AC with similarity to the prokaryotic CYTH domain proteins in its genome (Figures 1, 4). Since ACs can be activated by either intracellular or extracellular signals to produce cAMP, the signaling specificity for ACs warrants further investigation. Treatment of trophozoites with specific inhibitors of the key downstream effector proteins for cAMP: PKA (H-89) and EPAC (ESI-09) also resulted in reduced phagocytosis. All these evidences do support a traditional G-protein-regulated cAMP pathway in *Entamoeba*, but they do not deny the existence of a prokaryotic signaling route as well. We believe that our initial observations raise a new perspective on the signaling mechanisms operating in this protozoan. An in-depth functional characterization of these putative signaling pathway components will help better establish the role of cAMP signaling in parasite biology and pathogenesis.

## Data Availability Statement

All datasets presented in this study are included in the article/Supplementary Material.

## Author Contributions

SA conceived and designed the study. SA, PPR, and GA performed the experiments and wrote the paper. SA and SG contributed reagents, materials, and analysis tools. All authors contributed to the article and approved the submitted version.

## Conflict of Interest

The authors declare that the research was conducted in the absence of any commercial or financial relationships that could be construed as a potential conflict of interest.

## Supplementary Material

Supplementary Figure 1 Evolutionary relationship of Carbonic anhydrase. The evolutionary history of the enzyme carbonic anhydrase from *E. histolytica* was traced using the same enzyme from other organisms. The organisms included in the study were Hs, *Homo sapiens*; Dm, *Drosophila melanogaster*; Dd, *Dictyostelium discoideum*; Sc, *Saccharomyces cerevisiae*; Ld, *Leishmania donovani*. The amoebic enzyme (Blue) related more with the lower eukaryotes than with the human homolog.

Supplementary Figure 2 Erythrocyte uptake assay for phagocytosis. *E. histolytica* cells incubated with RBCs for the indicated time points (0–50 min) were assessed for RBC uptake by spectrophotometric analysis. The experiments were repeated independently three times in duplicate with error bars indicating the standard error.
