# Supplementary figures and images for: Uncovering the Cyclic AMP Signaling Pathway of the Protozoan Parasite Entamoeba histolytica and Understanding Its Role in Phagocytosis
Source: Front Cell Infect Microbiol. 2020 Sep 25;10:566726. doi: 10.3389/fcimb.2020.566726 (PMC7546249; doi:10.3389/fcimb.2020.566726)

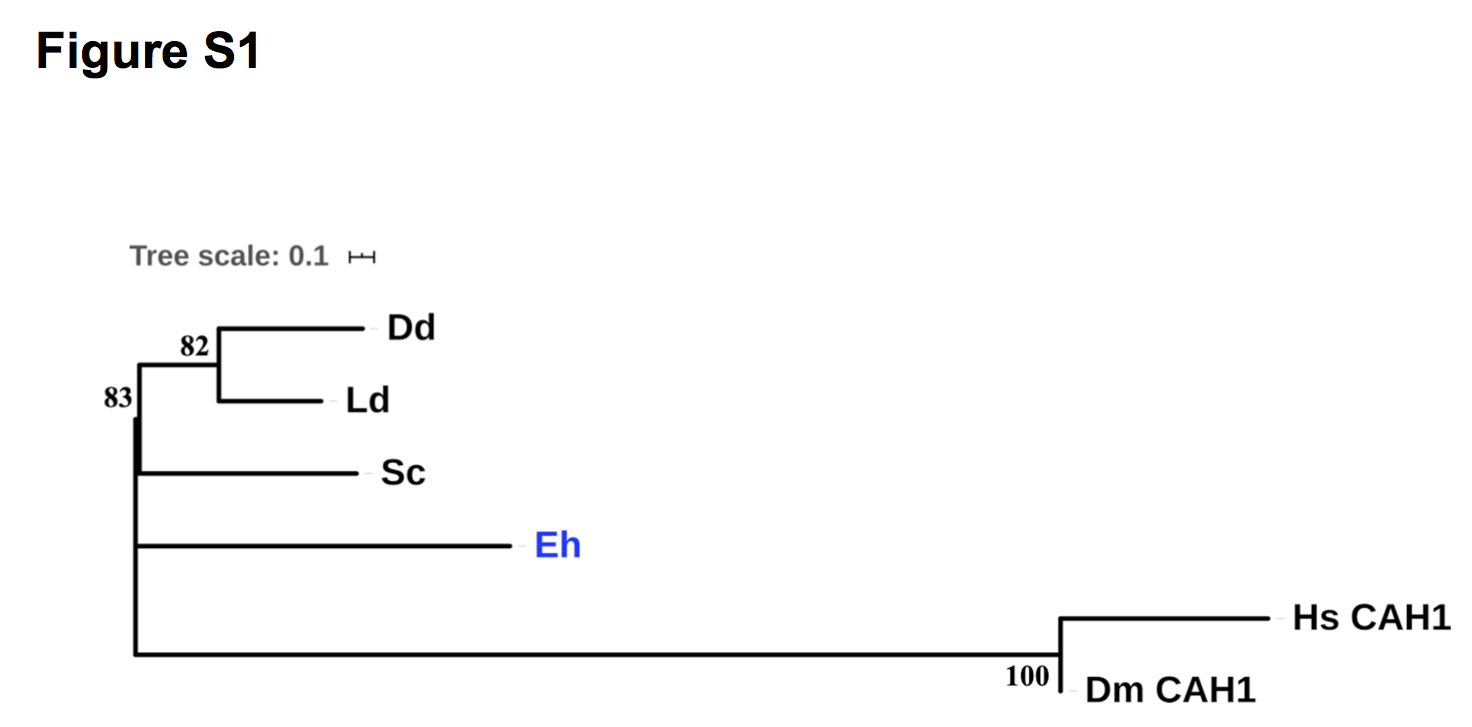

Supplement: Supplementary Figure 1 — Evolutionary relationship of Carbonic anhydrase. The evolutionary history of the enzyme carbonic anhydrase from E. histolytica was traced using the same enzyme from other organisms. The organisms included in the study were Hs, Homo sapiens; Dm, Drosophila melanogaster; Dd, Dictyostelium discoideum; Sc, Saccharomyces cerevisiae; Ld, Leishmania donovani. The amoebic enzyme (Blue) related more with the lower eukaryotes than with the human homolog. [file Image_1.TIFF]

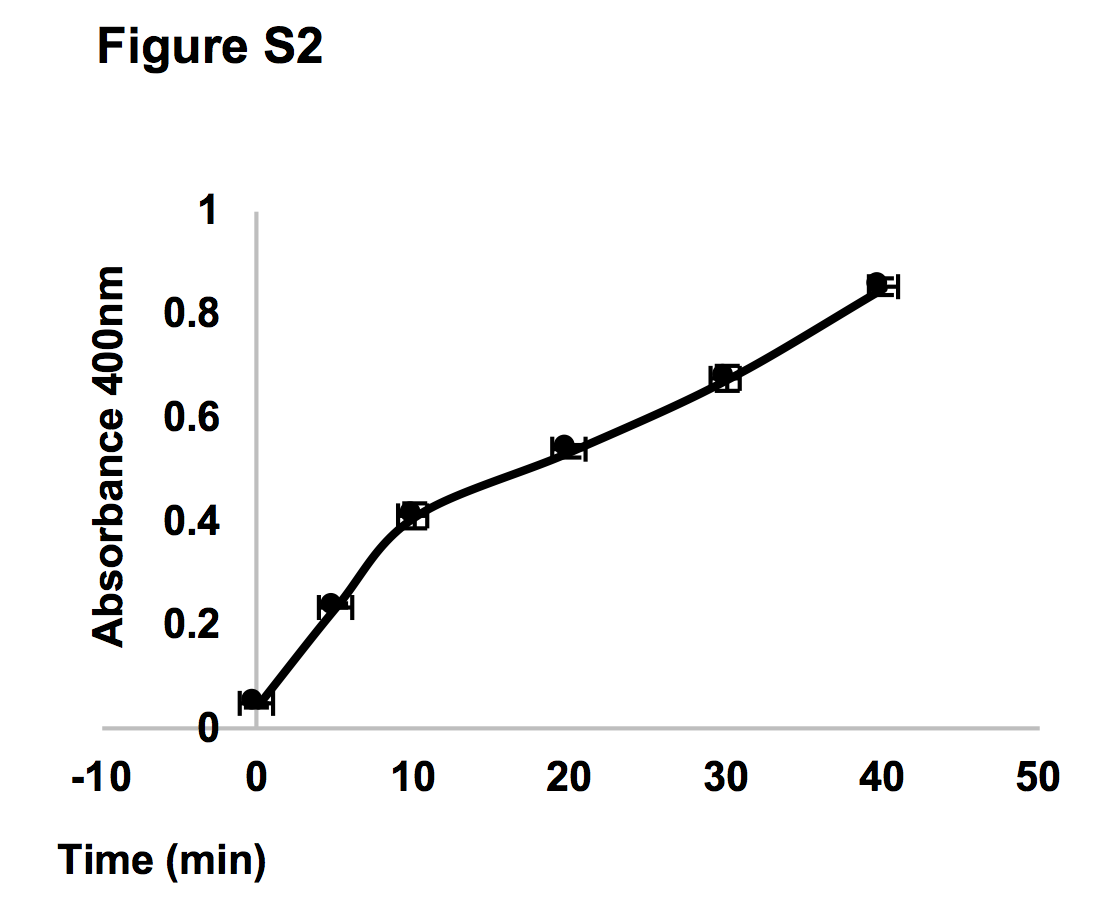

Supplement: Supplementary Figure 2 — Erythrocyte uptake assay for phagocytosis. E. histolytica cells incubated with RBCs for the indicated time points (0–50 min) were assessed for RBC uptake by spectrophotometric analysis. The experiments were repeated independently three times in duplicate with error bars indicating the standard error. [file Image_2.TIFF]
